# Using Moss to Assess Airborne Heavy Metal Pollution in Taizhou, China

**DOI:** 10.3390/ijerph14040430

**Published:** 2017-04-17

**Authors:** Xiaoli Zhou, Qin Chen, Chang Liu, Yanming Fang

**Affiliations:** Co-Innovation Center for Sustainable Forestry in Southern China, College of Biology and the Environment, Nanjing Forestry University, Nanjing 210037, China; zhouxiaoli0404@163.com (X.Z.); tzchenqin2010@163.com (Q.C.); liuchang890822@163.com (C.L.)

**Keywords:** air quality, heavy metals, moss biomonitoring, contamination factor, ecological risk assessment, factor analysis, GIS technology

## Abstract

Bryophytes act as bioindicators and bioaccumulators of metal deposition in the environment. To understand the atmospheric deposition of heavy metals (cadmium (Cd), chromium (Cr), copper (Cu), mercury (Hg), nickel (Ni), lead (Pb), and zinc (Zn)) in Taizhou, East China, samples of moss (*Haplocladium microphyllum*) were collected from 60 sites selected by a systematic sampling method during the summer of 2012, and the concentrations of these heavy metals were determined by inductively coupled plasma atomic emission spectroscopy (ICP-AES). The results suggested that the concentrations of these metals varied moderately among different sites, indicating a similar contamination level for each element throughout the monitoring region. The mean values under investigation were higher than those from neighboring cities, such as Wuxi, Xuzhou, and Nanjing, and much higher than those in Europe based on a 2010 survey. Significant (*p* < 0.01) correlations were identified among some of the heavy metals, suggesting that these originated from identical sources. There was no statistically significant correlation between Hg and all the other elements. Spatial distribution maps of the elements over the sampled territory were created using Arc-GIS 9.0. The potential ecological risk index indicated that the air was heavily polluted by Cd and Hg, and that there was a considerable potential ecological risk from all the heavy metals studied.

## 1. Introduction

Air pollution leads to seven million premature deaths annually worldwide, making poor air quality one of the most serious environmental health risks in the world [1]. It is associated with the input of heavy metals into vegetation and the surface soil, the resulting toxicity of which can harm public health [2,3]. Air quality has been monitored by applying different methods and models, such as sampling of bulk deposition or wet deposition samples, and by analyzing mosses, angiospermous leaves, or gymnospermous needles [4,5]. Detecting metal deposition by moss analysis is a suitable technique for monitoring atmospheric pollution [3,6,7]. Mosses lack a real root system or a well-developed cuticle layer [8,9], therefore, they absorb nutrients and heavy metals directly from atmosphere via dry, occult and wet atmospheric deposition over their entire surface [10].

Heavy metals distribute widely in the environment as a result of a range of human and natural sources. Environmental sources of pollutants include demolition and construction, farming, industrial development, mining and mineral processing, automobiles, windblown dust, and transport-related activities on roads [11]. Anthropogenic metal emissions represent a global environmental pollution problem. The annual global emissions of cadmium (Cd) and lead (Pb) reached 22,000 t and 783,000 t, respectively [12]. High enrichment factors of most trace metals in the western North Pacific Ocean were observed at a coastal station in Japan, and principal component analysis (PCA) results clearly indicated the effect of anthropogenic emissions [13]. In many cities in China, such as Beijing, Shanghai, Guangdong and Shenyang, the contents of lead (Pb) far exceed 50 mg·kg^−1^, which is the environmental quality recommended threshold in the European Union (EU) [14]. The toxicity of heavy metals depends on several factors including the dose, route of exposure, and chemical species. Numerous studies worldwide have confirmed that the main threats to human health from heavy metals are associated with exposure to lead (Pb), cadmium (Cd), chromium (Cr), mercury (Hg), and arsenic (As) because of their high degree of toxicity [15,16]. The technical deposition sampler has high purchasing costs, and its operation and the analysis of the resulting data are expensive. One instrument is not sufficient to resolve the varying nature of pollutant concentrations on spatial scales smaller than the size of a metropolitan area, such as at the neighborhood level or smaller. Measuring pollutant levels in bioindicators is less costly than using instruments, making it possible to collect the large number of samples needed to detect and quantify pollutants that disperse only short distances from their source. Since the 1970s in Europe, mosses have been frequently used in large-scale monitoring surveys, providing valuable information on the relative spatial and temporal changes and sources of trace metal deposition [17,18,19,20,21]. Regional moss surveys have also been conducted in other regions in Asia [22], Africa [23], and North America [24]. However, the monitoring of air quality using mosses in China has lagged behind that in Europe, and only a few studies have been published recently [25,26,27,28]. Thus, it would be beneficial to expand the use of mosses as a biomonitoring technique to trace the distribution and sources of heavy metals in China. The results of such monitoring experiments could then be used by Government sectors to implement measures to control air pollution.

The study area, Taizhou (approx. 5786.25 km^2^), is located in central Jiangsu Province at 32°1’–33°10’ N and 119°38’–120°32’ E. It is an important region of the Yangtze River Delta and is characterized by a north subtropical monsoon climate with distinct seasonal divisions. In recent decades, the region has developed rapidly, and currently comprises 34,000 industrial enterprises, of which more than 1083 are scale enterprises. Electrical, chemical, building material, pharmaceutical, textile and food industries are the main industries in Taizhou. However, these industrial activities have resulted in high levels of environment pollution in the territory.

The main purpose of this moss survey was to: (i) determine the levels of heavy metals (Cd, Cr, Cu, Ni, Pb, Zn, and Hg) throughout Taizhou; (ii) map the spatial distribution patterns of the seven heavy metals; (iii) evaluate the pollution level and ecological risk of each metal; and (iv) identify the local sources of each metal.

## 2. Materials and Methods

### 2.1. Sample Collection

The moss *Haplocladium microphyllum* was chosen as a biomonitor for several reasons. It is widespread across Jiangsu and can be found in all parts of the study area. The moss is characterized by a relatively large plant with creeping main stems and pinnately branching systems; it can be used repeatedly for urban ecological environment evaluations [29].

**Figure 1:**
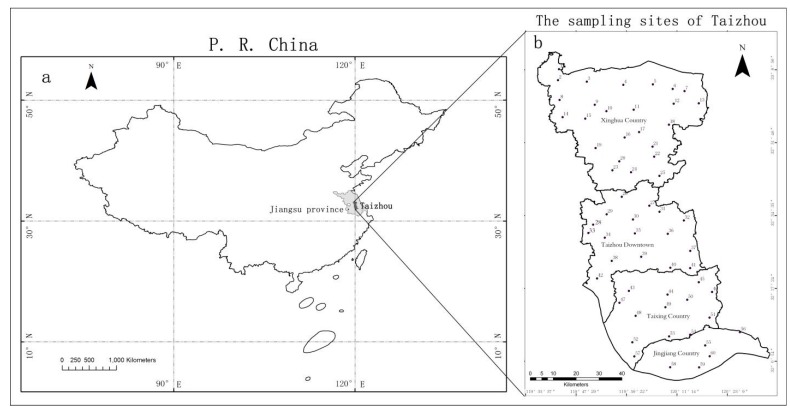
Location of Taizhou in China (**a**) and a map of Taizhou with the locations of the 60 moss-sampling sites (solid dots) (**b**). For the characteristics of each sample site, please refer to Table S1.

To achieve coverage of the whole survey area, the study region was divided into 10 km × 10 km grids, and moss samples were collected from a total of 60 sites (Figure 1, Table S1) from 10 August to 12 September 2012. The sampling sites were located at least 300 m from main roads, villages, and industrial areas, as well as at least 100 m from smaller roads and single houses [3]. Most moss samples were collected in open areas. At each of the 60 sites, five subsamples were collected and mixed to give one composite sample. Moss samples were picked up with a plastic shovel and stored in paper bags. During the sampling process, disposable polyethylene gloves were worn to avoid any contamination. The systematic sampling procedure was as described elsewhere [27,28].

### 2.2. Sample Preparation and Analysis

The green or greenish-brown parts of the plant were cleaned from extraneous litter and dead leaves with deionized water, and dried to a constant weight in a thermostatic dry machine for 48 h at 40 °C. The samples were then ground into powder under liquid nitrogen in a ceramic vessel. The powder was once again dried to constant weight at 40 °C and kept in a clean, dry paper bag. The use of metal equipment was avoided during the operation process to avoid affecting the results of the experimental measurements.

Approximately 0.5 g of each moss sample was transferred into Erlenmeyer flasks and wet digested at 120 °C with 10 mL mixed acid (HNO_3_:HClO_4_ = 4:1) until a transparent solution was obtained [30]. After cooling, the solution was transferred to a 25 mL volumetric flask that was then filled with deionized water. The presence and concentration of the heavy metals (Cd, Cr, Cu, Hg, Ni, Pb, and Zn) were determined by inductively coupled plasma atomic emission spectroscopy (ICP-AES; 4300DV, Perkin Elmer Corporation, Rodgau, Germany). The detection limits were 0.002 mg·kg^−1^ for Cu and Zn, 0.003 mg·kg^−1^ for Cd, Cr, and Ni, 0.005 mg·kg^−1^ for Pb, and 0.007 mg·kg^−1^ for Hg. The concentration of each element in the moss sample was corrected by subtracting blank values. The standard reference material, GBW10020 (Sigma-Aldrich, St. Louis, MO, USA), was analyzed to check the accuracy and precision of each metal analysis. The recovery percentages of heavy metals were between 95% and 105% for quantitative analysis. Three replicate measurements per moss sample were performed.

### 2.3. Data Analysis

The concentration data of the selected elements from moss samples across the 60 sampling sites were analyzed using SPSS 17.0 (IBM Corporation, Somers, NY, USA) for descriptive statistics. The statistical significance was based on an alpha of 0.05.

The geographic information system, Arc-GIS 9.0 (Esri Corporation, Tokyo, Japan), was used to generate the maps representing the geographical distribution of the pollution patterns of each element [31]. The maps were based on the mean values, and the data was transformed into a continuous surface by a universal Kriging interpolator technique. The maps revealed the spatial variation of each element. Semivariograms were used for spatial interpolation. This method is able to calculate the predictions from the measured points within neighborhoods. Cross-validation was then used to verify the credibility of each interpolation.

Contamination factor (CF) scales were used to determine the contamination level of each element [32]. The CF was calculated as the ratio of the mean value of each element from the moss samples from the study area (C_Mi_) versus the average value of the three sample sites showing the lowest concentration of the corresponding metal from the study area (C_Bi_; considered to be the background level) [32]. The CF values comprised six categories [33]: (1) C1: no contamination, CF < 1; (2) C2: suspected contamination, 1 ≤ CF ≤ 2; (3) C3: slight contamination, 2 < CF ≤ 3.5; (4) C4: moderate contamination, 3.5 < CF ≤ 8; (5) C5: serious contamination, 8 < CF ≤ 27; (6) C6: extreme contamination, CF > 27.

The potential ecological risk index (RI) was proposed to measure the overall ecological risk of multiple heavy metals [34]. The RI of all the heavy metals in a moss sample was calculated using Equation (1):

(1) $$\mathrm{RI}=\overset{n}{\underset{i=1}{\sum }}E_{r}^{i}$$

where *E_r_^i^* represents the potential ecologicalrisk coefficient of metal *i* and *n* was the number of heavy metals analyzed in the sample (*n* = 7 in present study).

The potential ecological risk coefficient (*E_r_^i^*) was calculated using Equation (2):

(2) $$E_{r}^{i}=T_{r}^{i}\times C_{f}^{i}=T_{r}^{i}\times \frac{C^{i}}{C_{n}^{i}}$$

where *T_r_^i^* was the toxic-response coefficient of a certain heavy metal (i.e., for Cd, Cr, Cu, Hg, Ni, Pb, and Zn 30, 2, 5, 40, 5, 5, and 1, respectively) [34,35]; *C_f_^i^* was the contamination coefficient of a certain heavy metal, calculated as the ratio of the measured concentration of HM i (*C^i^*) versus the background value of HM i (*C_n_^i^*). The classification criteria of pollution and potential ecological risk level was as follows: *E_r_^i^* < 40, low risk; 40 ≤ *E_r_^i^* < 80, moderate risk; 80 ≤ *E_r_^i^* < 160, considerable risk; 160 ≤ *E_r_^i^* < 320, very high risk; and *E_r_^i^* ≥ 320, dangerous; RI < 150, low risk; 150 ≤ RI < 300, moderate risk; 300 ≤ RI < 600, considerable risk; and RI ≥ 600, very high risk [34].

Multivariate data analysis is a powerful way of investigating multivariate and complex data sets by revealing trends and relationships of the parameters. Factor analysis (FA) is one such multivariate method and was used to obtain an overview of the data and to identify possible sources of heavy metals in the present study.

## 3. Results and Discussion

### 3.1. Quality Control of the Analysis

The quality control of ICP-AES (inductively coupled plasma atomic emission spectroscopy) results was checked by multiple analyses of the GBW10020 reference sample. The ICP-AES concentration data of the elements in the reference sample were in good agreement with the certified data (Table 1). The relative standard deviation (RSD) was between 0.5% and 7.1% based on 12 parallel determinations (Table 1). The results showed the good accuracy and precision of this method.

### 3.2. Heavy Metal Concentrations in the Moss Samples

The descriptive statistics of the seven heavy metals in the moss samples are shown in Table 2. These showed that the order of the heavy metals in terms of their average concentration across the study site was: Zn > Cu > Pb > Cr > Ni > Cd > Hg. All of the heavy metals under investigation showed moderate variation in concentration, with the coefficients of variation (CV) ranging from 25% to 75%. The largest CV value was recorded for Zn (73.8%), followed by Cr (58.3%), Cd (56.2%), Ni (51.6%), Cu (49.8%), and Hg (28.6%). High values of CV (>75%), Kurtosis (>3), Skewness (>0) are likely to indicate the influence of complicated factors on the concentrations of these heavy metals in mosses [36]. The moderate variation values in this study are also likely to reflect similar contamination levels for each element throughout the monitoring region. The European moss survey showed that the concentration level of Hg in moss samples remained stable for a long period of time [37]. Thus, the low value of CV in the current study for Hg could be related to the stability of Hg in moss.

The mean concentrations of the elements in moss samples from Taizhou were slightly higher than those from three neighboring cities in China for all the heavy metals, but were much higher than those recorded in Europe (Table 3). The substantial differences of mean concentrations in the heavy metals in Taizhou compared with Europe indicate the severe environmental pollution of the area studied, and the urgent need for the local Government to take effective measures to control and reduce this pollution.

### 3.3. Contamination Factors of Heavy Metals

Based on the CF results (Table 4), the study area was characterized by two categories of contamination scales: C3 and C4, described as slightly polluted and moderately polluted, respectively. The CF values for the heavy metals were higher in the Taizhou region compared with some European countries, such as Albania and Kosovo [3,39]. The evident differences in CF values between Taizhou and these two European countries suggest a high risk of heavy metal contamination in Taizhou.

### 3.4. Multivariate Analysis

The correlation data between the concentrations of the seven heavy metals are shown in Table 5: nine pairs of elements (Cd-Cu, Cd-Pb, Cd-Zn, Cr-Ni, Cr-Pb, Cu-Pb, Cu-Zn, Ni-P, and Pb-Zn) were significantly positively correlated, with the correlation coefficients for Pb-Cu, Pb-Cd, and Cr-Ni being >0.7. Significant correlations between elements within the same moss samples indicated that they came from the same pollution sources [40]. The significant positive correlations of Pb-Cu and Pb-Cd in the moss samples from the current study were in agreement with other similar investigations in China [27,28,38]. These correlations are likely to be related to traffic and industrial pollution [2]. There was no significant correlation between Hg and all the other elements. This could be related to the volatility of Hg: as a result of its physicochemical characteristics, which result in a long residence time in the atmosphere, Hg is a global pollutant.

To further analyze the correlation results, factor analysis was used. Two main factors were extracted from the results of the factor analysis and interpreted as source categories contributing to the element concentrations at the sampling sites. The results of the factor analysis and loading values of each element are shown in Table 6. Factor 1 was the strongest factor and explained 43.9% of the total variance. It was affected by high loadings of Cd, Cu, Pb, and Zn. The association of Cd, Cu, Pb, and Zn with the first factor is likely to be the result of anthropogenic activities (e.g., automobile exhaust and industrial emissions). Factor 2 represented 20.4% of the total variance, and was mainly influenced by high loads of Cr and Ni. This association is likely to be related to natural sources of these metals [41]. Although Hg clustered with Cr and Ni, the load value was only 0.370. This could be explained by the fact that Hg is not only influenced by industrial emissions, but also by coal-fired emissions [42].

### 3.5. Spatial Distribution of the Elements

**Figure 2:**
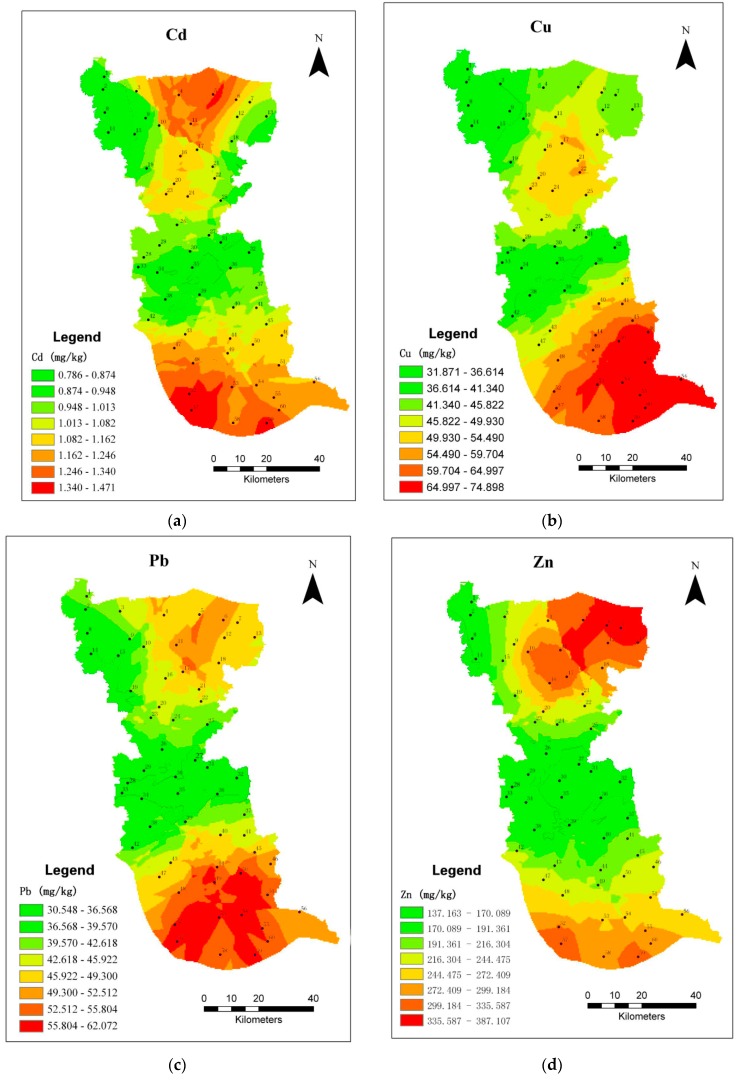

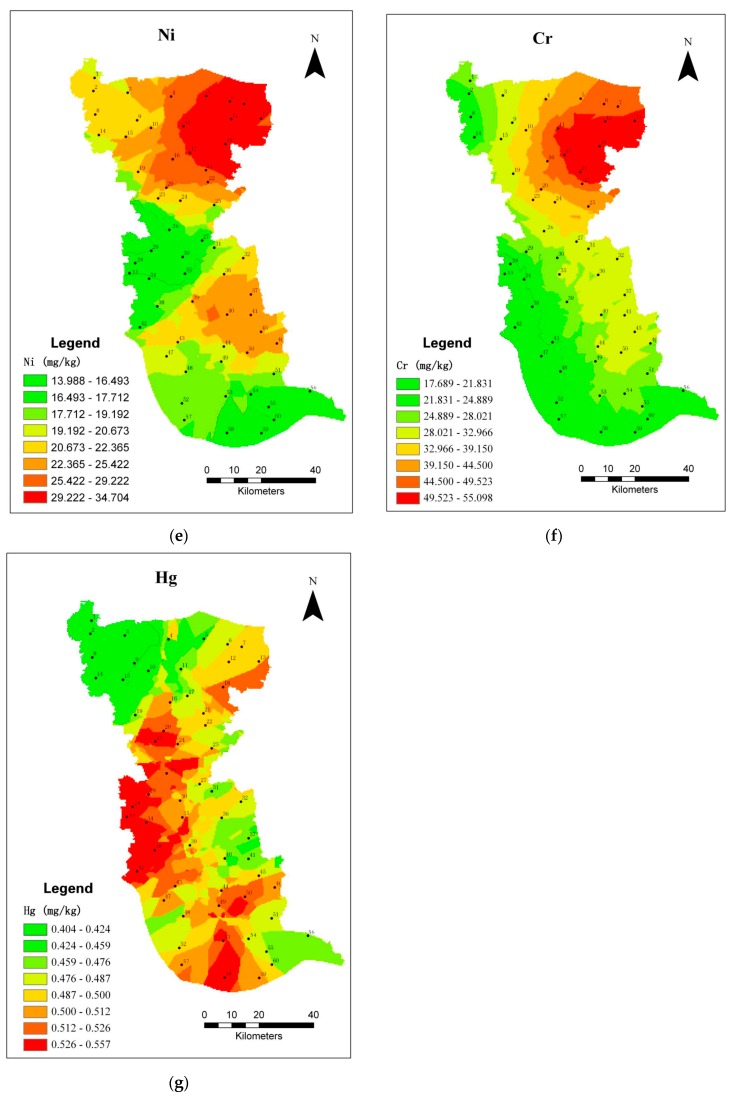
GIS maps of airborne trace metal deposition in Taizhou. (**a**) Distribution of Cd; (**b**) Distribution of Cu; (**c**) Distribution of Pb; (**d**) Distribution of Zn; (**e**) Distribution of Ni; (**f**) Distribution of Cr; (**g**) Distribution of Hg.

GIS maps were plotted to investigate the distribution of the elements in the moss samples (Figure 2).

#### 3.5.1. Cadmium, Copper, Lead, and Zinc

Cd, Cu, Pb, and Zn concentrations were higher in moss sampled from Taizhou compared with the mean values of neighboring cities in China and cities in Europe [21,25,27,28,38]. Manufacturing industries and construction were considered to be the main sources of Pb emission, whereas long-range transport was a secondary source of Pb emissions [29,43]. Cu was mostly associated with city dust, traffic exhaust, and soil [3]. Pb and Cu showed similar distribution patterns in the study area. The concentrations of these two elements in moss were generally high in the south and low in the central and northwest. In the south, county Jingjiang is close to the Yangtze River, which has numerous ports in important industrial cities in southern Jiangsu Province. The machinery manufacturing industry has developed in the south of the study area and the Ning-Tong highway runs across this region. Thus, industrial emissions, road transport, and long-range transport could be responsible for these high contents of Pb and Cu. A sub-top Pb level was recorded in northeast Taizhou and was likely to be from traffic emission because of the G204 National Road that passes along the northeast boundary of the study site. The next most-Cu-polluted areas were around sites 17, 22, and 25 (Figure 2), close to the largest stainless steel industrial area in China.

Cd and Zn concentrations were mostly affected by Zn smelters [44]. The highest concentrations of Cd and Zn were found in the south and north of the study region. The highest Zn-polluted and the second most-Cd-polluted regions were in the north, where there are several Zn refineries, such as those at Xinghong (site 5, Figure 2), Hongsheng (site 6, Figure 2), and Keda (site 7, Figure 2). The main contributors of Zn and Cd in the south, where the second most-Zn-polluted and highest Cd-polluted regions occurred, were likely to be a Zn refinery (site 52, Figure 2), and a nonferrous metal smelter (site 57, Figure 2), both in Taixing.

#### 3.5.2. Chromium and Nickel

The concentration of Cr increased from the southwest to the northeast of the study site. The highest Ni-polluted areas were in the north, similar to Cr, and central Taizhou (southeast of the Jiangyan district and northeast of the Taixing district). Cr and Ni generally occur in soil and can be emitted into the air as windblown particles. Northern Taizhou (Xinghua Country) is a predominantly agricultural area, where the use of pesticides and fertilizers could also lead to contamination of the soil by Cr and Ni. The high percentage of land use has been identified as the local main sources of Cr and Ni. The high concentrations of Ni in central Taizhou are likely to be associated with the exploitation of mineral resources.

#### 3.5.3. Mercury

The regional distribution of Hg was patchy and non-uniform. Hg is a global pollutant, and its levels in Taizhou were higher compared with those in Europe, including Kosovo [3,21]. Hg contamination was present in most areas of Taizhou, being particularly high in western and southern Taizhou (Figure 2). The sampling sites 34 and 42 were positioned close to the power plants of Guodian and the Merlin Chemical Group Ltd. (Taizhou, China), which are both large coal-fired enterprises. Combustion facilities have been identified as the main local source of Hg emissions. The next highest Hg level, in southern Taizhou, is likely to be the result of emissions from plastic factories and electrical plants.

### 3.6. Assessment of Heavy Metal Pollution and Potential Ecological Risk

*E_r_^i^* and RI (Table 7) were analyzed to further investigate the degree of heavy metal contamination and ecological hazards in Taizhou. As shown in Table 7, the mean values of *E_r_^i^* for the heavy metals were ranked in the following order: Cd > Hg > Cu > Pb > Ni > Zn > Cr. The mean values of *E_r_^i^* for most of the heavy metals (Cu, Pb, Ni, Zn, and Cr) were < 40, suggesting a low ecological risk. However, Hg had a higher mean value (135.17), indicating considerable ecological risk, and Cd had the highest mean value (250.89), demonstrating very high ecological risk.

Overall, the RI values ranged from 76.99 to 1002.16, with an average of 413.69 (Table 6), indicating a considerable potential ecological risk for all seven heavy metals. Cd and Hg were the main contributors to potential ecological risk in Taizhou, with a percentage of 60.65% and 32.67%, respectively, representing 93.92% of the total proportion. As shown in Table 8, low potential ecological risk was found in only one sampling site (site 18), moderate ecological risk in nine sites, considerable potential ecological risk in 43 sites, and very high ecological risk in seven sites (sites 5, 21, 41, 52, 55, 57, and 59). The low potential ecological risk site was located in northern Taizhou, whereas four of the seven very high ecological risk sites were located in southern Taizhou, indicating the severe effects of the industrial activities on atmospheric environmental quality in this part of the study site. 

## 4. Conclusions

Detecting heavy metal concentrations in samples of moss is a valuable method for monitoring the quality of the atmospheric environment. Spatial patterns of heavy metal concentrations in moss are metal specific, reflecting local variation in heavy metal deposition. The mean concentration levels of the heavy metals in moss samples from the current study were higher than the mean concentration levels in moss samples from Wuxi, Xuzhou, and Nanjing in China, and in sites in Europe. These data revealed a strong and complex effect of anthropogenic factors, such as coal-fired industrial plants, traffic emissions, and other industrial activities, combined with wind-blown soil dust on the levels of heavy metals in Taizhou.

*E_r_^i^* demonstrated that the study areas were highly or considerably polluted with Cd and Hg. RI indicated that the southern part of Taizhou was more contaminated than the other areas of the study site. The result of the zone risk index (RI = 413.69 > 300) showed that the whole of the study site was moderately polluted by the seven metals. Based on these results, further measures are required in Taizhou to decrease emissions of heavy metals from coal-fired industrial plants, industry, and transportation, to reduce the health risk to humans and the environment. FA is also a useful tool for identifying the causes of air pollution. In the current study, FA revealed traffic emissions, industrial emissions, coal-fired industrial plants, and natural sources as the main sources of atmospheric deposition in Taizhou.

## Figures and Tables

**Table 1:** Results of accuracy experiments by GBW10020 and precision experiment (*n* = 12).

Element	Certified Value (μg·g^−1^)	Obtained Value (μg·g^−1^)	RSD (%)
Cd	0.17	0.16	4.4
Cr	1.25	1.23	1.8
Cu	6.6	6.3	1.8
Hg	150	147	1.4
Ni	1.1	1.2	4.9
Pb	9.7	9.3	0.73
Zn	18	18	2.5

**Table 2:** Summary of heavy metal concentrations in mosses across Taizhou (mg·kg**^−^**^1^, DW)

Parameter	Cd	Cr	Cu	Hg	Ni	Pb	Zn
Minimum	0.05	6.55	14.48	0.13	5.85	8.75	63.28
Maximum	3.50	83.65	116.55	0.79	48.07	89.45	1096.25
Range	3.45	77.1	102.07	0.66	42.22	80.7	1032.97
Median	0.93	26.05	40.89	0.47	17.79	39.18	198.86
Mean	1.05	30.92	48.00	0.49	20.95	44.18	232.83
SD	0.59	18.03	23.91	0.14	10.82	21.64	171.84
CV%	56.2	58.3	49.8	28.6	51.6	49.0	73.8
Skewness	1.36	1.35	0.08	−0.03	0.93	0.55	2.70
Kurtosis	3.74	1.48	−0.12	−0.39	−0.13	−0.70	10.54

DW: Dry Weight.

**Table 3:** Comparison of the mean concentrations of heavy metals from mosses in Taizhou, neighboring cities in China, and Europe (mg·kg**^−^**^1^).

Elements	Taizhou (Current Study)	Wuxi [28]	Xuzhou [27]	Nanjing [38]	Europe (2010) [21]
Cd	1.05	0.93	0.82	0.53	0.19
Cr	30.92	15.1	26.3	8.2	1.50
Cu	48.0	36.5	28.0	17.7	6.99
Ni	20.95	10.1	20.1	5.5	1.82
Pb	44.18	31.2	33.0	21.3	3.69
Zn	232.83	206.3	155	74.9	32.9
Hg	0.49	-	-	-	0.05

**Table 4:** The contamination factors (CFs) and contamination classification [33].

Parameter	Cd	Cr	Cu	Hg	Ni	Pb	Zn
CF	5.25	3.78	3.02	2.30	3.04	3.60	3.54
Classification	C4	C4	C3	C3	C3	C4	C4
Contamination	Moderate	Moderate	Slightly	Slightly	Slightly	Moderate	Moderate

**Table 5:** Pearson correlation coefficients between element concentrations in moss samples from Taizhou.

Elements	Cd	Cr	Cu	Hg	Ni	Pb
Cr	0.163	-	-	-	-	-
Cu	0.669 **	0.243	-	-	-	-
Hg	0.087	0.217	0.056	-	-	-
Ni	0.152	0.709 **	0.197	0.105	-	-
Pb	0.710 **	0.344 **	0.739 **	0.168	0.342 **	-
Zn	0.399 **	0.22	0.377 **	0.204	0.218	0.400 **

Cell contents: Person correlation, ** < 0.01.

**Table 6:** Results of the factor analysis.

Variable	Factor 1	Factor 2
Cd	0.885	0.021
Cr	0.137	0.901
Cu	0.873	0.094
Hg	0.096	0.370
Ni	0.116	0.880
Pb	0.858	0.267
Zn	0.565	0.239
Variance (%)	0.439	0.204

**Table 7:** Potential ecological risk assessment of heavy metals in Taizhou following atmospheric deposition.

Elements	*E_r_^i^*		Ecological Risk Level
	Range	Mean	
Cd	11.90–833.33	250.89	very high
Cr	0.17–2.15	0.79	low
Cu	3.25–26.13	10.76	low
Hg	36.82–219.38	135.17	considerable
Ni	1.10–9.00	3.92	low
Pb	1.67–17.07	8.43	low
Zn	1.01–17.51	3.72	low
RI	76.99–1002.16	413.69	considerable

**Table 8:** Summary of the potential ecological risk assessment of heavy metals.

Sampling	RI	Ecological Risk Level	Sampling	RI	Ecological Risk Level	Sampling	RI	Ecological Risk Level
1	318.37	considerable	21	718.52	very high	41	604.85	very high
2	206.89	moderate	22	389.10	considerable	42	386.39	considerable
3	433.78	considerable	23	452.68	considerable	43	479.84	considerable
4	398.21	considerable	24	420.14	considerable	44	575.87	considerable
5	1002.16	very high	25	354.30	considerable	45	356.64	considerable
6	445.11	considerable	26	366.93	considerable	46	392.78	considerable
7	335.97	considerable	27	479.28	considerable	47	545.06	considerable
8	386.12	considerable	28	597.02	considerable	48	272.84	moderate
9	200.23	moderate	29	544.00	considerable	49	414.05	considerable
10	530.62	considerable	30	323.09	considerable	50	447.52	considerable
11	440.77	considerable	31	302.40	considerable	51	338.45	considerable
12	259.17	moderate	32	274.23	moderate	52	651.51	very high
13	351.77	considerable	33	303.85	considerable	53	396.90	considerable
14	174.91	moderate	34	278.91	moderate	54	525.51	considerable
15	316.33	considerable	35	372.57	considerable	55	655.96	very high
16	578.96	considerable	36	425.53	considerable	56	389.70	considerable
17	401.93	considerable	37	323.95	considerable	57	665.82	very high
18	76.99	low	38	318.73	considerable	58	216.36	moderate
19	349.57	considerable	39	313.43	considerable	59	777.54	very high
20	354.72	considerable	40	290.80	moderate	60	316.00	considerable

RI: the potential ecological risk index.

## Supplementary Materials

The following are available online at www.mdpi.com/1660-4601/14/4/430/s1, Figure S1: The Semivariogram Modeling and Cross Validation for Cd, Figure S2: The Semivariogram Modeling and Cross Validation for Cr, Figure S3: The Semivariogram Modeling and Cross Validation for Cu, Figure S4: The Semivariogram Modeling and Cross Validation for Hg, Figure S5: The Semivariogram Modeling and Cross Validation for Ni, Figure S6: The Semivariogram Modeling and Cross Validation for Pb, Figure S7: The Semivariogram Modeling and Cross Validation for Zn, Table S1: Sampling sites, geographical coordinates, slope, distance from sea and aspect of the monitoring stations in Taizhou, China.

## References

[1] World Health Organization (2014). Burden of Disease from Household Air Pollution for 2012.

[2] Han Y.M., Du P.X., Cao J.J., Posmentier E.S. (2006). Multivariate analysis of heavy metal contamination in urban dusts of Xi’an, Central China. Sci. Total Environ..

[3] Maxhuni A., Lazo P., Kane S., Qarri F., Marku E., Harmens H. (2016). First survey of atmospheric heavy metal deposition in Kosovo using moss biomonitoring. Environ. Sci. Pollut. Res..

[4] Nickel S., Schröder W. (2016). Integrative evaluation of data derived from biomonitoring and models indicating atmospheric deposition of heavy metals. Environ. Sci. Pollut. Res..

[5] Becker K., Schroeter-Kermani C., Seiwert M., Rüther M., Conrad A., Schulz C., Wilhelm M., Wittsiepe J., Günsel A., Dobler L. (2013). German health-related environmental monitoring: Assessing time trends of the general population’s exposure to heavy metals. Int. J. Hyg. Envirion. Health.

[6] Markert B., Herpin U., Berlekamp J., Oehlmann J., Grodzinska K., Mankovska B., Suchara I., Siewers U., Weckert V., Lieth H. (1996). A comparison of heavy metal deposition in selected Eastern European countries using the moss monitoring method, with special emphasis on the “Black Triangle”. Sci. Total Environ..

[7] Harmens H., Norris D.A., Steinnes E., Kubin E., Piispanen J., Alber R., Aleksiayenak Y., Blum O., Coşkun M., Dam M. (2010). Mosses as biomonitors of atmospheric heavy metal deposition: spatial patterns and temporal trends in Europe. Environ. Pollut..

[8] Fernández J.A., Ederra A., Núñez E., Martínez-Abaigar J., Infante M., Heras P., Elías M.J., Mazimpaka V., Carballeira A. (2002). Biomonitoring of metal deposition in northern Spain by moss analysis. Sci. Total Environ..

[9] Blagnyte R., Paliulis D. (2011). Research into heavy metals pollution of atmosphere applying moss as bioindicator: A literature review. Environ. Res. Eng. Manag..

[10] Broady P.A., Liggett D., Storey B., Cook Y. (2015). Life on land. Exploring the Last Continent.

[11] Melaku S., Morris V., Raghavan D., Hosten C. (2008). Seasonal variation of heavy metals in ambient air and precipitation at a single site in Washington, DC. Environ. Pollut..

[12] Pérez A.L., Anderson K.A. (2009). DGT estimates cadmium accumulation in wheat and potato from phosphate fertilizer applications. Sci. Total Environ..

[13] Okubo A., Takeda S., Obata H. (2013). Atmospheric deposition of trace metals to the western North Pacific Ocean observed at coastal station in Japan. Atmos. Res..

[14] Chamseddine M., Wided B.A., Guy H., Marie-Edith C., Fatma J. (2009). Cadmium and copper induction of oxidative stress and antioxidative response in tomato (*Solanum lycopersicon*) leaves. Plant Growth Regul..

[15] Järup L. (2003). Hazards of heavy metal contamination. Br. Med. Bull..

[16] Bradl H.B. (2005). Heavy Metals in the Environment: ORIgin, Interaction and Remediation.

[17] Rühling Å., Rasmussen L., Pilegaard K., Makinen A., Steinnes E. (1987). Survey of atmospheric heavy metal deposition in the Nordic countries in 1985—Monitored by moss analysis. Nord.

[18] Rühling Å. (1994). AtmospheRIc Heavy Metal Deposition in Europe—Estimation Based on Moss Analysis.

[19] Rühling Å., Steinnes E. (1998). Atmospheric Heavy Metal Deposition in Europe 1995–1996.

[20] Harmens H., Ilyin I., Mills G., Aboal J.R., Alber R., Blum O., Coşkun M., De Temmerman L., Fernández J.Á., Figueira R. (2012). Country-specific correlations across Europe between modelled atmospheric cadmium and lead deposition and concentrations in mosses. Environ. Pollut..

[21] Harmens H., Norris D.A., Sharps K., Mills G., Alber R., Aleksiayenak Y., Blum O., Cucu-Man S.M., Dam M., De Temmerman L. (2015). Heavy metal and nitrogen concentrations in mosses are declining across Europe whilst some “hotspots” remain in 2010. Environ. Pollut..

[22] Saxena D.K., Singh S., Srivastava K. (2008). Atmospheric heavy metal deposition in Garhwal hill area (India): Estimation based on native moss analysis. Aerosol Air Qual. Res..

[23] Olajire A.A. (1998). A survey of heavy metal deposition in Nigeria using the moss monitoring method. Environ. Int..

[24] Schilling J.S., Lehman M.E. (2002). Bioindication of atmospheric heavy metal deposition in the Southeastern US using the moss *Thuidium delicatulum*. Atmos. Environ..

[25] An L., Cao T., Yu Y.H. (2006). Bryophytes and environmental heavy metal pollution monitoring. Chin. J. Ecol..

[26] Cao T., An L., Wang M., Lou Y., Yu Y., Wu J., Zhu Z., Qing Y., Janice G. (2008). Spatial and temporal changes of heavy metal concentrations in mosses and its indication to the environments in the past 40 years in the city of Shanghai, China. Atmos. Environ..

[27] Liu C., Zhou P., Fang Y.M. (2016). Monitoring airborne heavy metal using mosses in the City of Xuzhou, China. Bull. Environ. Contam. Toxicol..

[28] Yan Y., Zhang Q., Wang G.G., Fang Y.M. (2016). Atmospheric deposition of heavy metals in Wuxi, China: Estimation based on native moss analysis. Environ. Monit. Assess..

[29] Harmens H., Norris D.A., Koerber G.R., Buse A., Steinnes E., Rühling Å. (2008). Temporal trends (1990–2000) in the concentration of cadmium, lead and mercury in mosses across Europe. Environ. Pollut..

[30] Wei S., Zhou Q., Koval P.V. (2006). Flowering stage characteristics of cadmium *hyperaccumulator Solanum. nigrum* L. and their significance to phytoremediation. Sci. Total Environ..

[31] Finkelshtein M.Y., Deev K.V. (1999). GIS-INTEGRO a set of tools for development of information system for nature management. Geoinformatika.

[32] Fernández J.A., Carballeira A. (2001). A comparison of indigenous mosses and topsoils for use in monitoring atmospheric heavy metal deposition in Galicia (northwest Spain). Environ. Pollut..

[33] Shakya K., Chettri M.K., Sawidis T. (2014). Use of mosses for the survey of heavy metal deposition in ambient air of the Kathmandu valley applying active monitoring technique. Ecoprint Int. J. Ecol..

[34] Hakanson L. (1980). An ecological risk index for aquatic pollution control. A sedimentological approach. Water Res..

[35] Xu Z.Q., Ni S.J., Tuo X.G. (2008). Calculation of heavy metals toxicity coefficient in the evaluation of potential ecological risk index. Environ. Sci. Technol..

[36] Allajbeu S., Yushin N.S., Qarri F., Duliu O.G., Lazo P., Frontasyeva M.V. (2016). Atmospheric deposition of rare earth elements in Albania studied by the moss biomonitoring technique, neutron activation analysis and GIS technology. Environ. Sci. Pollut. Res..

[37] Harmens H., Mills G., Hayes F., Norris D. Air Pollution and Vegetation: ICP Vegetation Annual Report 2010/2011. http://nora.nerc.ac.uk/15072/1/ICP_Vegetation_annual_report_2010-11.pdf.

[38] Wang A.X. (2010). Mosses and Trees as Indicators for Heavy Metal Pollution in the Atmosphere of Nanjing City, China. PhD Thesis.

[39] Qarri F., Lazo P., Stafilov T., Frontasyeva M., Harmens H., Bekteshi L., Baceva K. (2014). Multi-elements atmospheric deposition study in Albania. Environ. Sci. Pollut. Res..

[40] Huang Y.J., Liu D.Y., Wang Y.B. (2006). Heavy metals accumulation by bryophytes. Chin. J. Ecol..

[41] Zhang M., Hao W. (2009). Concentrations and chemical forms of potentially toxic metals in road-deposited sediments from different zones of Hangzhou. China J. Environ. Sci..

[42] UNEP/AMAP, Expert Group (2008). Assessment Programme (AMAP) and United Nations Environment Programme (UNEP): Technical Background Report to the Global Atmospheric Mercury Assessment.

[43] Coskun M., Cayir A., Coskun M., Kilic O. (2011). Heavy metal deposition in moss samples from East and South Marmara Region, Turkey. Environ. Monit. Assess..

[44] Berg T., Røyset O., Steinnes E., Vadset M. (1995). Atmospheric trace element deposition: principal component analysis of ICP-MS data from moss samples. Environ. Pollut..

