# Supplementary material for: Using Moss to Assess Airborne Heavy Metal Pollution in Taizhou, China
Source: Int J Environ Res Public Health. 2017 Apr 17;14(4):430. doi: 10.3390/ijerph14040430 (PMC5409631; doi:10.3390/ijerph14040430)
Supplement: Supplementary file 1 [file ijerph-14-00430-s001.pdf]

Table S1. Sampling sites, geographical coordinates, slope, distance from sea and aspect of the monitoring stations in Taizhou, China

Sampling	Latitude N	Longitude E	Altitude(m)	Slope	Distance From Sea(km)	Aspect	Sampling	Latitude N	Longitude E	Altitude(m)	Slope	Distance From Sea(km)	Aspect
1	33°09′04.2″	119°43′29.7″	4.68	10.79‰	97.89	East	31	32°35′27.2″	120°07′09.3″	0.97	1.12‰	78.68	Northeast
2	33°06′28.6″	119°43′15.7″	0.63	3.71‰	100.07	North	32	32°33′24.6″	120°12′53.3″	5.86	14.08‰	70.23	Northwest
3	33°06′07.6″	119°50′01.2″	3.19	13.32‰	90.61	West	33	32°30′27.6″	119°50′23.4″	0.16	7.68‰	105.89	East
4	33°05′22.8″	119°58′37.1″	2.81	1.61‰	78.78	Southeast	34	32°29′21.0″	119°54′16.4″	5.55	15.99‰	100.25	Northeast
5	33°05′29.5″	120°05′35.9″	1.5	12.34‰	68.66	South	35	32°30′21.6″	120°01′21.1″	5.44	1.60‰	89.00	North
6	33°04′24.8″	120°10′16.9″	1.89	2.77‰	62.70	South	36	32°30′15.2″	120°09′06.8″	7.46	3.08‰	77.12	South
7	33°03′54.6″	120°13′02.7″	1.6	2.06‰	59.20	Northwest	37	32°26′14.6″	120°14′24.8″	7	2.74‰	71.19	Northwest
8	33°01′49.8″	119°43′37.7″	0.53	3.36‰	102.86	Southeast	38	32°23′53.1″	119°55′54.0″	3.74	15.49‰	100.15	West
9	33°00′41.1″	119°51′54.4″	1.88	10.06‰	91.77	Southeast	39	32°24′51.6″	120°02′50.9″	5.79	2.96‰	89.20	South
10	32°59′10.4″	119°54′39.6″	1.11	2.98‰	88.89	Northwest	40	32°22′15.1″	120°09′42.6″	6.3	3.58‰	80.75	East
11	32°59′31.0″	120°01′04.4″	3.83	3.46‰	79.53	East	41	32°22′11.8″	120°14′23.7″	6.36	6.99‰	74.08	Northeast
12	33°00′56.5″	120°10′30.0″	1.45	1.39‰	65.27	Southwest	42	32°19′45.7″	119°52′27.3″	4.75	3.40‰	107.67	Northeast
13	33°01′02.5″	120′16′24.2″	2	0.33‰	57.31	East	43	32°16′49.5″	119°59′56.2″	5.5	1.24‰	98.85	North
14	32°57′46.3″	119°44′19.9″	3.44	11.55‰	104.74	Southwest	44	32°15′56.7″	120°09′03.4″	3.29	5.47‰	86.86	Northwest
15	32°57′25.7″	119°49′40.3″	1.64	15.11‰	97.30	Southeast	45	32°18′51.5″	120°16′24.2″	4.91	8.92‰	74.18	East
16	32°52′58.1″	119°58′56.9″	2.32	20.21‰	88.13	Southeast	46	32°16′33.9″	120°19′32.6″	5.08	1.10‰	72.35	Northwest
17	32°54′14.9″	120°02′20.9″	3.49	9.96‰	82.36	East	47	32°14′00.9″	119°57′43.8″	5.01	6.40‰	104.24	East
18	32°55′59.7″	120°09′22.2″	3.62	12.80‰	70.94	Northwest	48	32°10′56.5″	120°01′31.4″	3.74	22.17‰	101.76	South
19	32°50′28.5″	119°52′06.9″	1.64	1.98‰	99.68	Northwest	49	32°12′58.6″	120°08′32.7″	4.29	6.59‰	90.40	Southwest
20	32°47′21.8″	119°57′41.0″	2.29	1.95‰	93.05	West	50	32°14′43.5″	120°13′41.1″	7.8	5.45‰	81.86	Northeast
21	32°50′48.8″	120°05′30.4″	2.18	12.31‰	79.52	Southwest	51	32°10′31.9″	120°18′56.6″	6.25	4.70‰	80.04	Southeast
22	32°48′27.8″	120°05′53.8″	3.19	3.76‰	80.09	Northeast	52	32°04′41.1″	120°00′45.6″	6.5	3.38‰	109.16	Southeast

23	32°45′14.5″	119°56′03.7″	3.58	9.96‰	96.12	Southwest	53	32°06′05.9″	120°09′24.6″	7.24	10.49‰	96.78	West
24	32°44′46.0″	120°00′27.6″	2.26	10.28‰	89.22	Southwest	54	32°06′29.9″	120°14′22.1″	4.29	2.76‰	90.38	Southeast
25	32°43′55.8″	120°07′08.0″	0.78	5.52‰	78.72	East	55	32°03′57.4″	120°17′55.8″	6.21	21.74‰	89.66	Southwest
26	32°39′00.0″	119°58′15.4″	1.56	5.25‰	92.29	Northwest	56	32°07′05.7″	120°26′05.8″	3.81	7.16‰	76.59	South
27	32°36′53.9″	120°04′45.0″	0.35	1.79‰	82.25	Northeast	57	32°01′22.3″	120°01′13.2″	3.33	4.47‰	112.30	Northwest
28	32°32′25.1″	119°51′31.8″	2.7	13.22‰	103.62	North	58	31°58′49.2″	120°09′40.8″	4.1	7.15‰	105.53	Southeast
29	32°34′51.1″	119°54′42.7″	3.02	11.50‰	98.19	North	59	31°58′47.1″	120°16′29.8″	0.93	1.96‰	98.15	Southeast
30	32°33′39.1″	120°00′54.3″	1.02	4.80‰	88.76	West	60	32°01′25.4″	120°18′58.4″	2.72	12.56‰	91.93	Northeast

The GIS maps of airborne trace metal deposition were generated by a Kriging interpolator technique. We calculated semi-variograms before applying Kriging interpolation. The cross validation was used to evaluate the accuracy of the model. Kriging provides a number of functions for empirical semi-variational function modeling. The commonly used functions are Circular, Spherical, Exponential, Gaussian, and Stable. In the comparison of the models obtained for different functions, we can refer to several indicators in Prediction Error. The Model that meets the following criteria are optimal: Mean Standardized is closest to 0, Root-Mean-Square is the smallest, and Average Standard Error is closest to 1. The five functions mentioned above were used for semi-variograms modeling of each element and we chose a more appropriate model for interpolation of each element with cross validation. If we show the semi-variant model of each function and the cross validation results, the content is too much. The semi-variograms of optimal model as well as the cross-validation results would be included into this supplement.

(1) The five functions mentioned above were used for semi-variograms modeling of Cd. According to the criteria mentioned previously, the Stable was a more appropriate model for interpolation of Cd with cross validation. The Semivariogram Modeling and Cross Validation for Cd were shown in Fig S1. The sill is the partial sill plus the nugget. The nugget, sill and nugget/sill are 0.26286, 0.42027, and 0.62545, respectively.

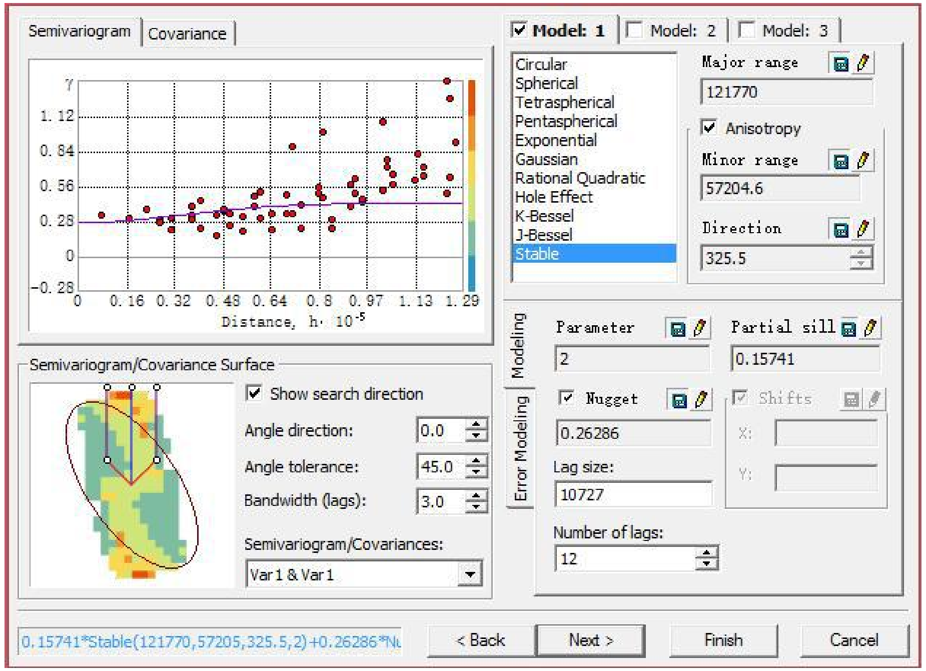

(a) The Semivariogram Modeling for Cd

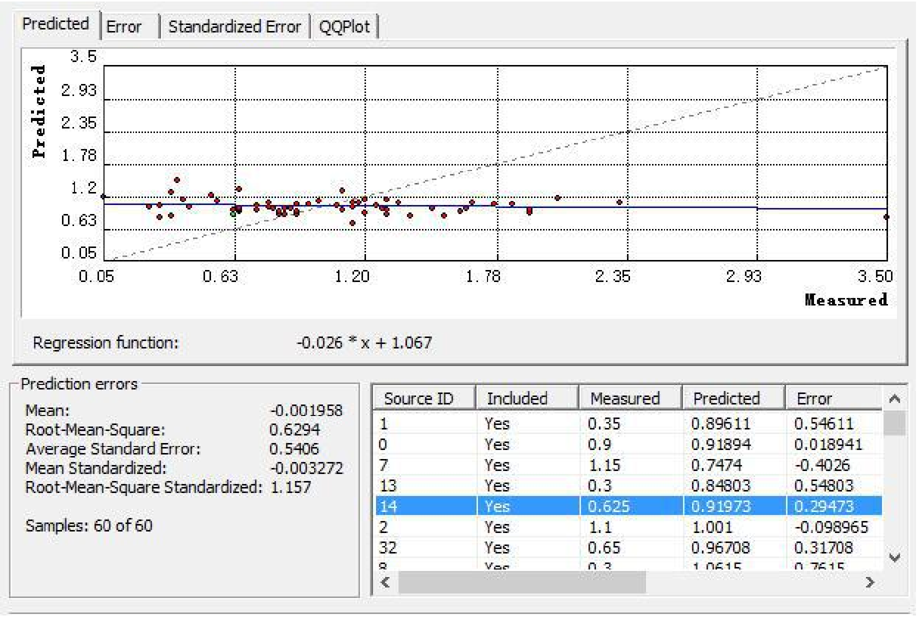

(b) The Cross Validation for Cd

Fig S1. The Semivariogram Modeling and Cross Validation for Cd

(2) The five functions mentioned above were used for semi-variograms modeling of Cr. According to the criteria mentioned previously, the Gaussian was a more appropriate model for interpolation of Cr with cross validation. The Semivariogram Modeling and Cross Validation for Cr were shown in Fig S2. The sill is the partial sill plus the nugget. The nugget, sill and nugget/sill are 220.51, 408.18, and 0.5402, respectively.

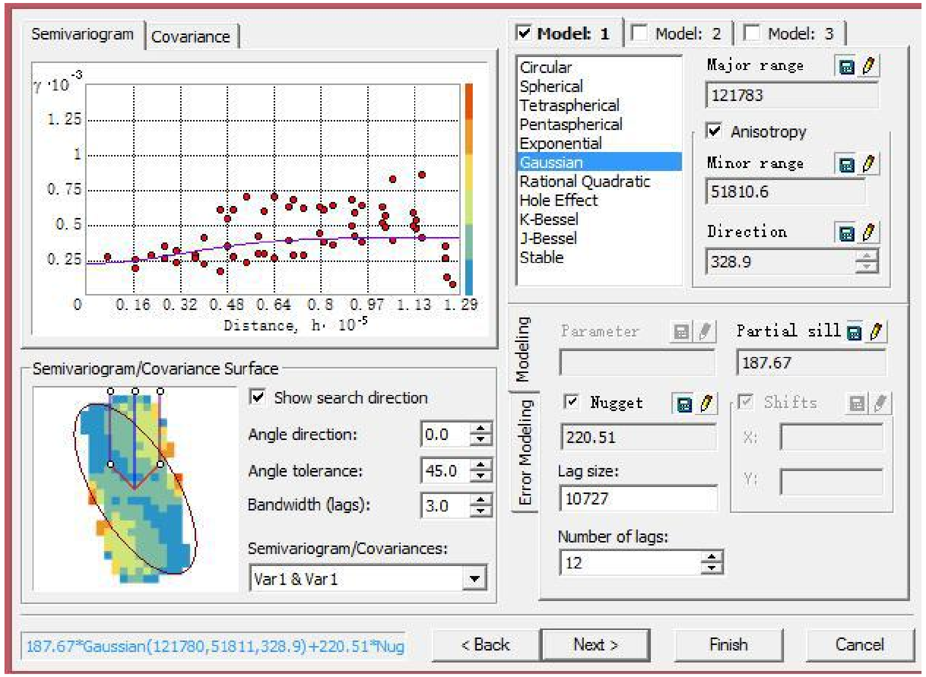

(a) The Semivariogram Modeling for Cr

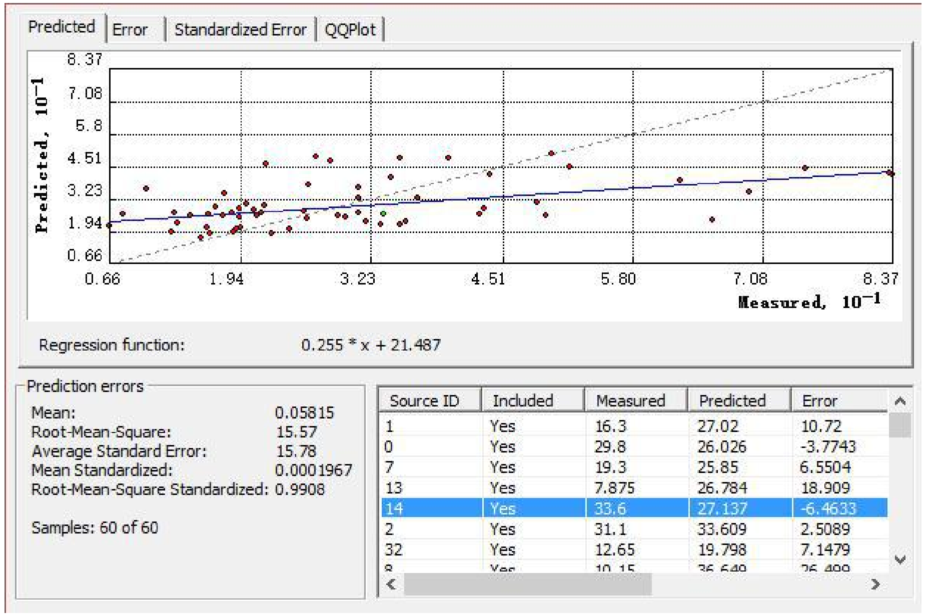

(b) The Cross Validation for Cr

Fig S2. The Semivariogram Modeling and Cross Validation for Cr

(3) The five functions mentioned above were used for semi-variograms modeling of Cu. According to the criteria mentioned previously, the Exponential was a more appropriate model for interpolation of Cu with cross validation. The Semivariogram Modeling and Cross Validation for Cu were shown in Fig S3. The sill is the partial sill plus the nugget. The nugget, sill and nugget/sill are 419.12, 628.24, and 0.6671, respectively.

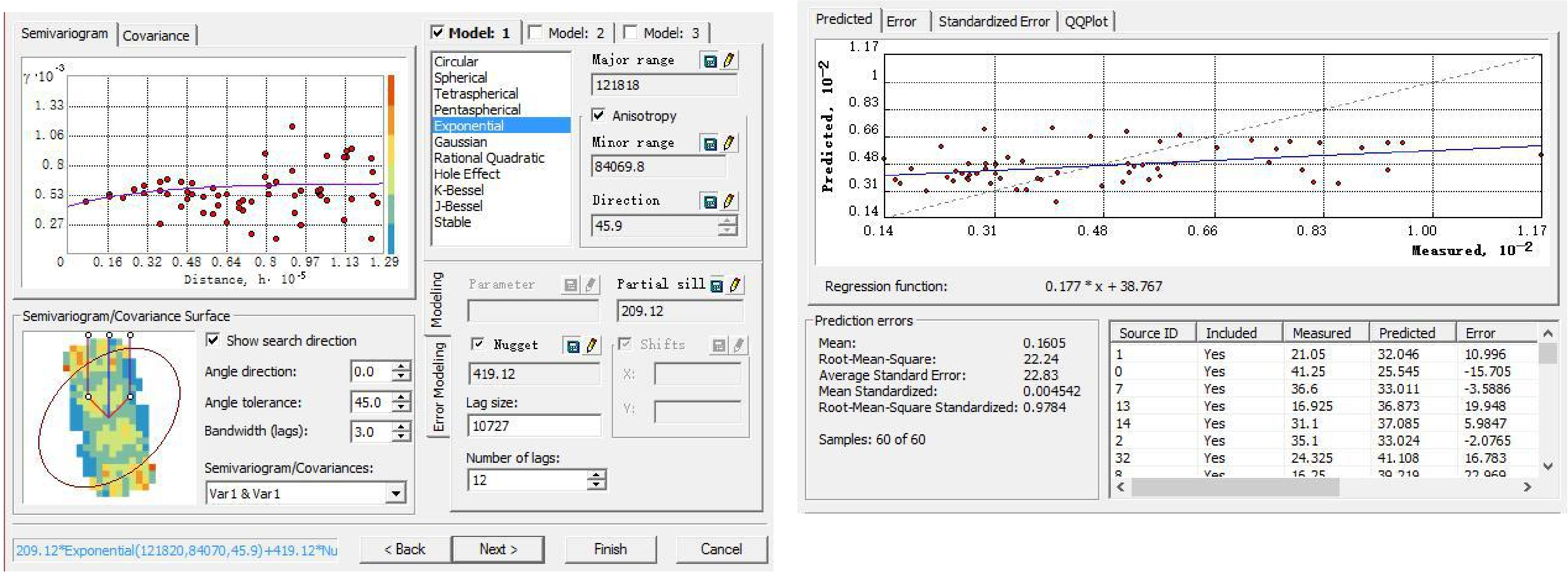

(a) The Semivariogram Modeling for Cu

(b) The Cross Validation for Cu

Fig S3. The Semivariogram Modeling and Cross Validation for Cu

(4) The five functions mentioned above were used for semi-variograms modeling of Hg. According to the criteria mentioned previously, the Exponential was a more appropriate model for interpolation of Hg with cross validation. The Semivariogram Modeling and Cross Validation for Hg were shown in Fig S4. The sill is the partial sill plus the nugget. The nugget, sill and nugget/sill are 0.0056708, 0.0195268, and 0.2904, respectively.

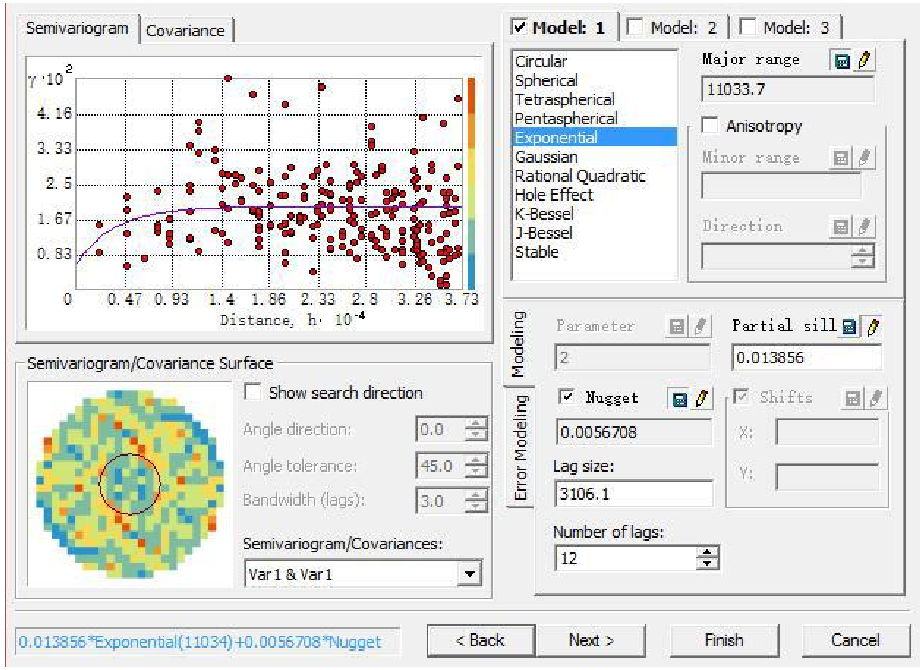

(a) The Semivariogram Modeling for Hg

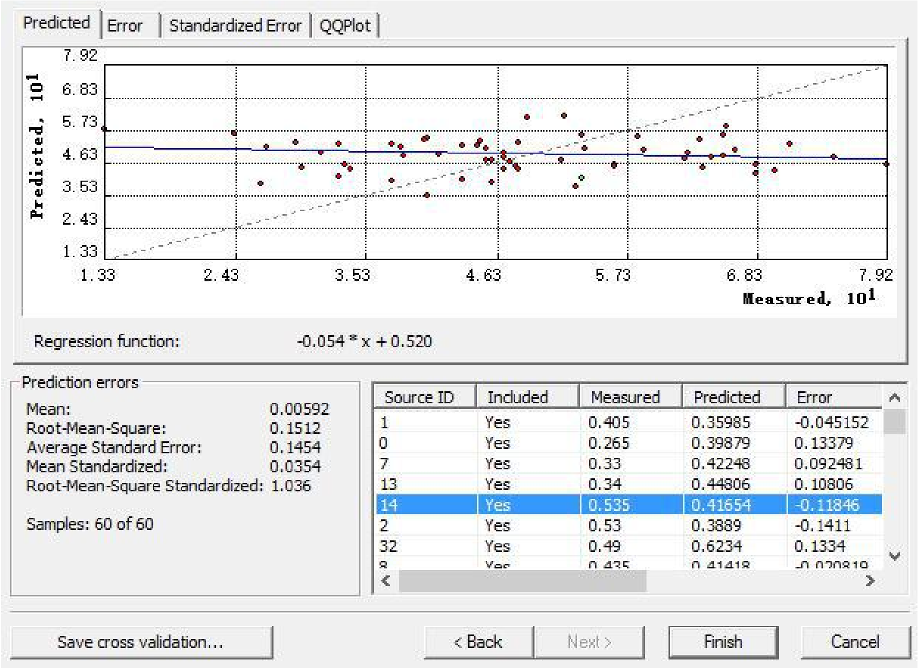

(b) The Cross Validation for Hg

Fig S4. The Semivariogram Modeling and Cross Validation for Hg

(5) The five functions mentioned above were used for semi-variograms modeling of Ni. According to the criteria mentioned previously, the Gaussian was a more appropriate model for interpolation of Ni with cross validation. The Semivariogram Modeling and Cross Validation for Ni were shown in Fig S5. The sill is the partial sill plus the nugget. The nugget, sill and nugget/sill are 101.72, 121.434, and 0.8376, respectively.

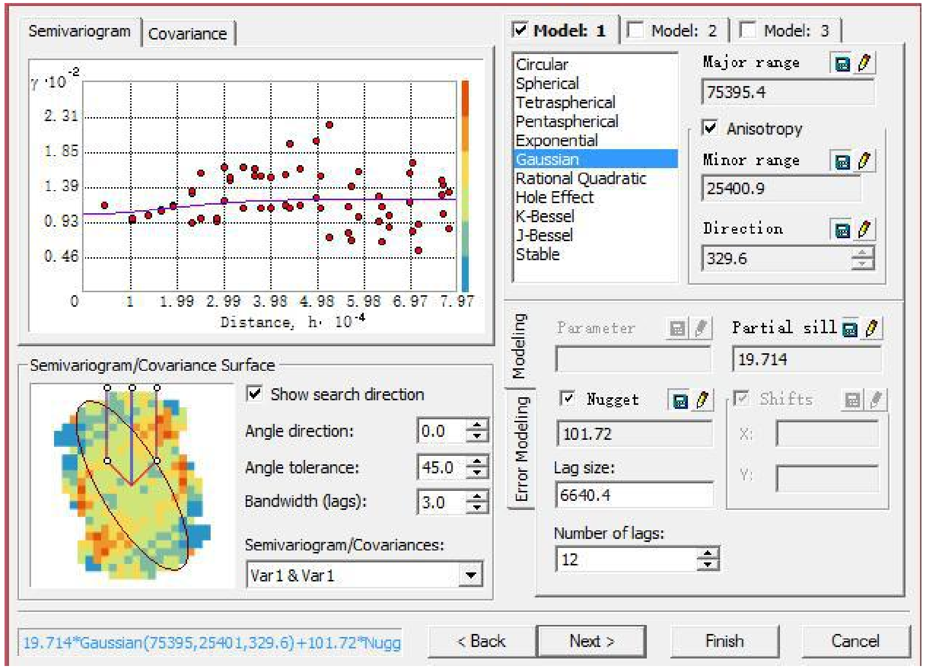

(a) The Semivariogram Modeling for Ni

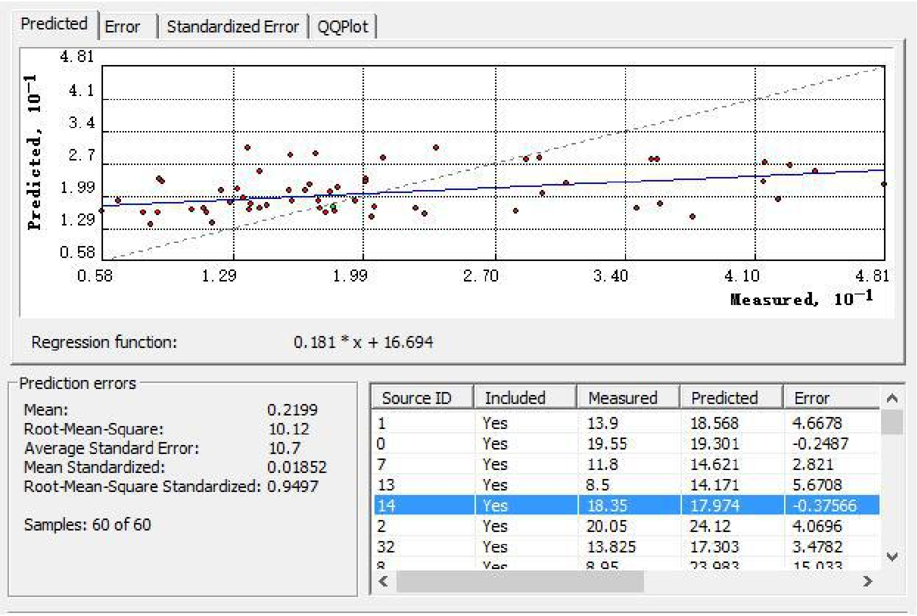

(b) The Cross Validation for Ni

Fig S5. The Semivariogram Modeling and Cross Validation for Ni

(6) The five functions mentioned above were used for semi-variograms modeling of Pb. According to the criteria mentioned previously, the Gaussian was a more appropriate model for interpolation of Pb with cross validation. The Semivariogram Modeling and Cross Validation for Pb were shown in Fig S6. The sill is the partial sill plus the nugget. The nugget, sill and nugget/sill are 412.34, 539.06, and 0.7649, respectively.

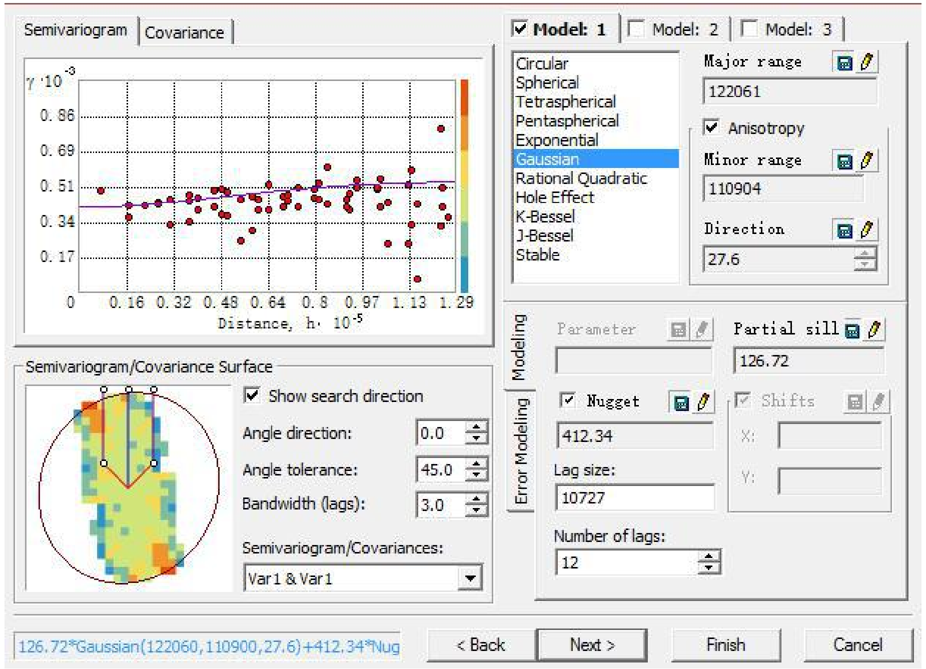

(a) The Semivariogram Modeling for Pb

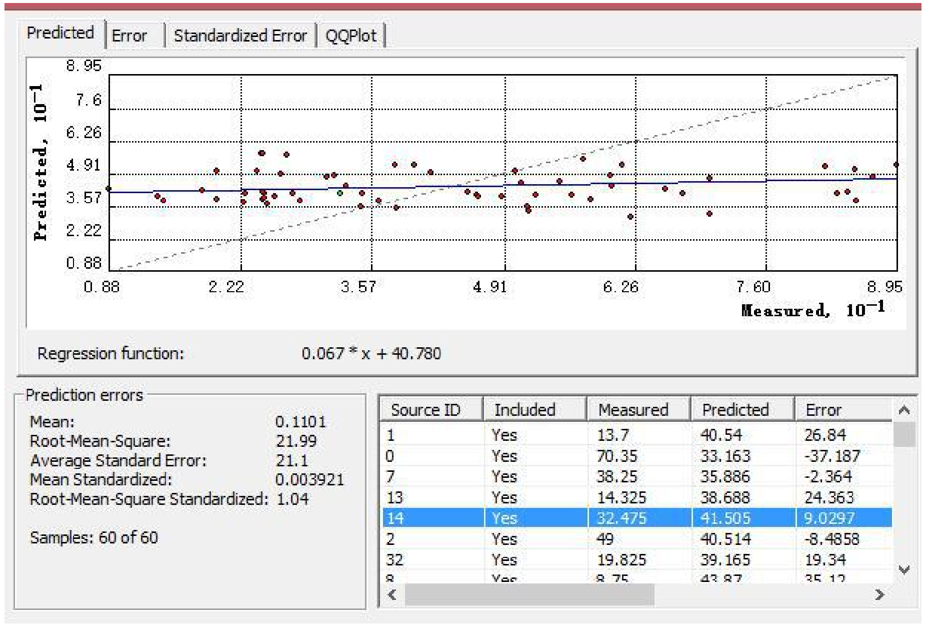

(b) The Cross Validation for Pb

Fig S6. The Semivariogram Modeling and Cross Validation for Pb

(7) The five functions mentioned above were used for semi-variograms modeling of Zn. According to the criteria mentioned previously, the Stable was a more appropriate model for interpolation of Zn with cross validation. The Semivariogram Modeling and Cross Validation for Zn were shown in Fig S7. The sill is the partial sill plus the nugget. The nugget, sill and nugget/sill are 19413, 35693, and 0.5439, respectively.

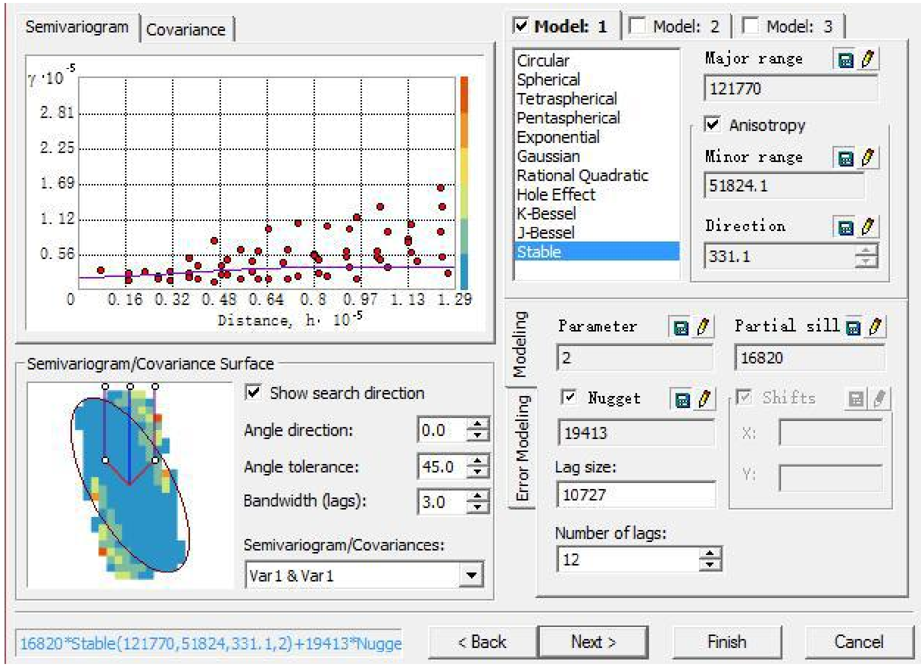

(a) The Semivariogram Modeling for Zn

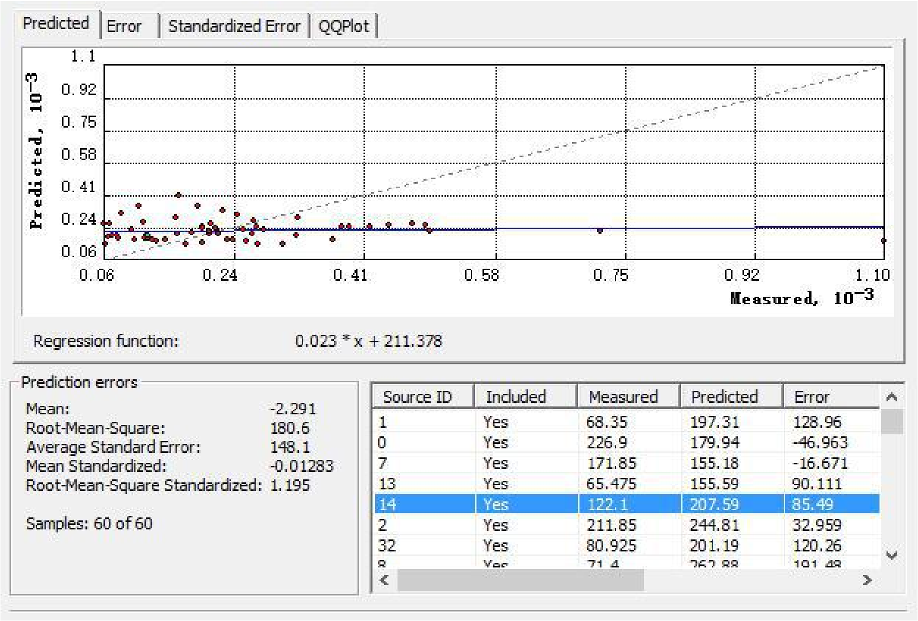

(b) The Cross Validation for Zn

Fig S7. The Semivariogram Modeling and Cross Validation for Zn
